# Analysis of Epidemiological and Clinical Characteristics of COVID-19 in Northwest Mexico and the Relationship Between the Influenza Vaccine and the Survival of Infected Patients

**DOI:** 10.3389/fpubh.2021.570098

**Published:** 2021-03-25

**Authors:** Uriel A. Angulo-Zamudio, Francisco M. Martínez-Villa, Nidia Leon-Sicairos, Hector Flores-Villaseñor, Jorge Velazquez-Roman, Abraham Campos-Romero, Jonathan Alcántar-Fernández, Francisco Urrea, Secundino Muro-Amador, Julio Medina-Serrano, Jesus J. Martinez-Garcia, Jaime Sanchez-Cuen, Jorge Angulo-Rocha, Adrian Canizalez-Roman

**Affiliations:** ^1^Centro de Investigación Aplicada a la Salud Pública (CIASaP), School of Medicine, Autonomous University of Sinaloa, Culiacan, Mexico; ^2^Programa de Maestría en Ciencias en Biomedicina Molecular, Autonomous University of Sinaloa (UAS), Culiacan, Mexico; ^3^Unidad de Medicina Familiar No. 46, Instituto Mexicano del Seguro Social (IMSS), Culiacan, Mexico; ^4^Pediatric Hospital of Sinaloa, Culiacan, Mexico; ^5^The Sinaloa State Public Health Laboratory, Secretariat of Health, Culiacan, Mexico; ^6^Salud Digna A.C., Culiacán, Mexico; ^7^Hospital Regional, Instituto de Seguridad y Servicios Sociales de los Trabajadores del Estado, Culiacán, Mexico; ^8^Coordinación en Investigación en Salud, Órgano de Operación Administrativa Desconcentrada (OOAD), Instituto Mexicano del Seguro Social (IMSS), Culiacan Sinaloa, Mexico; ^9^The Women's Hospital, Secretariat of Health, Culiacan, Mexico

**Keywords:** COVID-19, Mexico, influenza vaccine, clinical characteristics, epidemiological

## Abstract

The first cases of unexplained pneumonia were reported in Wuhan, China, in December of 2019. Later, a novel coronavirus (SARS-CoV-2) was identified as the causal agent of pneumonia. This virus has since spread to more than 180 countries and has been declared a pandemic by the World Health Organization. Herein, we aimed to determine the epidemiological and clinical characteristics of symptomatic patients with coronavirus disease 2019 (COVID-19) and the relationship between the influenza vaccine with a lower risk of severe COVID-19 infection in the state of Sinaloa. We collected demographic and clinical data of 4,040 patients with acute respiratory infections across Sinaloa state hospitals from February 28 to May 15, 2020. The prevalence of COVID-19 among hospitalized patients with respiratory symptoms in Sinaloa showed 45.2% of men were more affected than women (*p* < 0.001), and people aged 40–49 years were the most affected. The main symptoms of COVID-19 infection were cough and fever (*p* < 0.001), while hypertension, obesity, and type 2 diabetes were the chronic diseases associated with COVID-19 than non-COVID-19 (*p* < 0.003). Healthcare workers were most likely to be infected compared to other occupations (*p* < 0.001). The general lethality rate was 14.1%, and males >62 years were the ones who had a higher lethality rate (*p* < 0.001); the aforementioned chronic diseases were related to higher lethality of COVID-19 (*p* < 0.001). Likewise, higher lethality was seen in housewives and patient retirees/pensioners compared with other occupations (*p* < 0.001). Finally, we found there was a relationship between influenza vaccination and a lower risk of severe COVID-19 infection and mortality (*p* < 0.001). These findings showed that healthcare workers, men >62 years with chronic diseases, and retired people were most affected. Furthermore, the influenza vaccine could decrease the severeness of COVID-19 cases.

## Introduction

Coronavirus belongs to a family of viruses that cause symptoms related to respiratory diseases. Although these viruses predominantly have animal hosts, they can be transmitted to other species via different mechanisms to infect new hosts, including humans. In December 2019, Wuhan, China, reported the first cases of pneumonia caused by an unknown pathogen. Later, on December 30, 2019, a novel virus was isolated from the bronchoalveolar lavage fluid of patients with acute pneumonia in the Jinyintan Hospital (1).

Soon after, the World Health Organization (WHO) identified a novel coronavirus (2019-nCOV) as a causal agent of pneumonia reported in China (2). This new coronavirus was identified as Severe Acute Respiratory Syndrome Coronavirus 2 (SARS-CoV-2) by the International Committee on Taxonomy of Viruses (3). SARS-CoV-2 is highly contagious and the causative agent for coronavirus disease 2019 (COVID-19). Globalization and international mobility facilitated the spread of the disease to more than 160 countries, leading to more than 179,112 infections and 7,426 deaths; accordingly, the WHO declared COVID-19 as a pandemic on March 11, 2020 (4).

COVID-19 has also affected Sinaloa; this state housed the first COVID-19 diagnosed case in Mexico on February 28, 2020. The case was a 41-year-old man who had traveled to Italy. This person was confined to a hotel until he recovered from the infection. After that, COVID-19 spread to the rest of Mexico.

The importance of this study in Sinaloa lies in the large proportion of the population that suffers from chronic diseases, such as obesity, type 2 diabetes, and hypertension. In fact, Sinaloa is one of the 10 main states with obesity and diabetes cases in Mexico (5). The association among chronic diseases and COVID-19 severe infection is well-studied (6). Mexico is one of the main countries with obesity worldwide; Sinaloa is one of the states with a higher burden of this disease in the country, and the new COVID-19 disease could thus affect the population more in this state. Here, we reported the prevalence and epidemiological and clinical characteristics of COVID-19 cases in Sinaloa: one of the first states in Mexico with reported SARS-CoV-2 infections. We also analyzed the association between comorbidities and non-communicable diseases (NCD) with COVID-19 lethality, taking into account their high burden in Mexico. Finally, we analyzed the relationship between influenza vaccination and survival among patients with COVID-19.

## Materials and Methods

### Region of Study

This study was carried out in Sinaloa State, located in the Northwest of Mexico. This state has a population of 2,966,321 subjects. In all, 50.6% (1,502,236) of them are women and 49.4% (1,464,085) are male. In terms of age, 44.8% (1,330,837) of the population in Sinaloa are between 0 and 24 years old, and 33.4% (992,097) are 40–85 years old (Instituto Nacional de Estadistica y Geografia, www.inegi.org.mx).

### Source of Data

Demographic data (sex, age, social security, and occupation), clinical information (comorbidities, signs and symptoms, history of influenza vaccination, and clinical outcome) from patients who sought care for respiratory symptoms, and the result of SARS-CoV-2 polymerase chain reaction (PCR) testing were collected between February 28 (date of first COVID-19 case in Sinaloa) and May 15, 2020; we identified 6,933 patients from different health institutions of Sinaloa state. Among them, the Mexican Social Security Institute (Instituto Mexicano del Seguro Social, IMSS), Hospitals of Health Services Sinaloa (Servicios de Salud, SSA), Institute of Security and Social Services for State Workers (Instituto de Seguridad y Servicios Sociales de los Trabajadores del Estado, ISSSTE), private hospitals, and hospitals from other government agencies were included in this cross-sectional and exploratory study.

COVID-19 was confirmed based on positive qRT-PCR results for SARS-CoV-2 and upon presentation of characteristic symptoms of the disease, whereas influenza H1N1, respiratory syncytial virus, influenza A H3 and B, enteroviruses, and rhinovirus infections were diagnosed according to Manual for the Laboratory Diagnosis and Virological Surveillance of Influenza and the Institute of Epidemiological Diagnosis and Reference (Instituto de Diagnóstico y Referencia Epidemiológicos, InDRE) standards (7). Patients negative for SARS-CoV-2 but who were affected by other respiratory viruses were classified as non-COVID-19.

Of 6,933 patients who sought care for respiratory symptoms (inclusion criteria), 4,040 were tested by qRT-PCR to identify SARS-CoV-2 infection. Samples that did not meet quality standards or triage criteria (symptoms of COVID-19) were excluded (*n* = 2,893).

### Statistical Analysis

Pearson's chi-square tested differences between groups and categories (age-group, sex, PCR test result, symptoms, clinical outcome, comorbidities, vaccination history, occupation, lethality, or survival).

Pearson's chi-square was also used to compare the effects of underlying diseases, prior vaccinations, and occupation on lethality. Moreover, prior vaccination was analyzed by a multivariate test, which was adjusted by sex. A *p* ≤ 0.05 was considered to indicate statistical significance; a Bonferroni correction was applied to avoid alpha 1 error. The data were analyzed using the statistical package SPSS® Statistics version 24 (IBM Corp., Armonk, NY, USA); the graphs were constructed with the SigmaPlot version 12 program (SYSTAT, CA, USA).

### Ethical Approval

The study was approved by the Ethics Committee of The Women's Hospital, Secretariat of Health (No. 202005-03) and was conducted following the ethical principles of the World Medical Association Declaration of Helsinki. Patients' personal identities and other private information were anonymized; therefore, the need for informed consent was waived in accordance with local legislation and national guidelines (NOM-012-SSA3-2012).

## Results

### Characteristics of Study Population and Distribution of COVID-19 Cases

**Figure 1:**
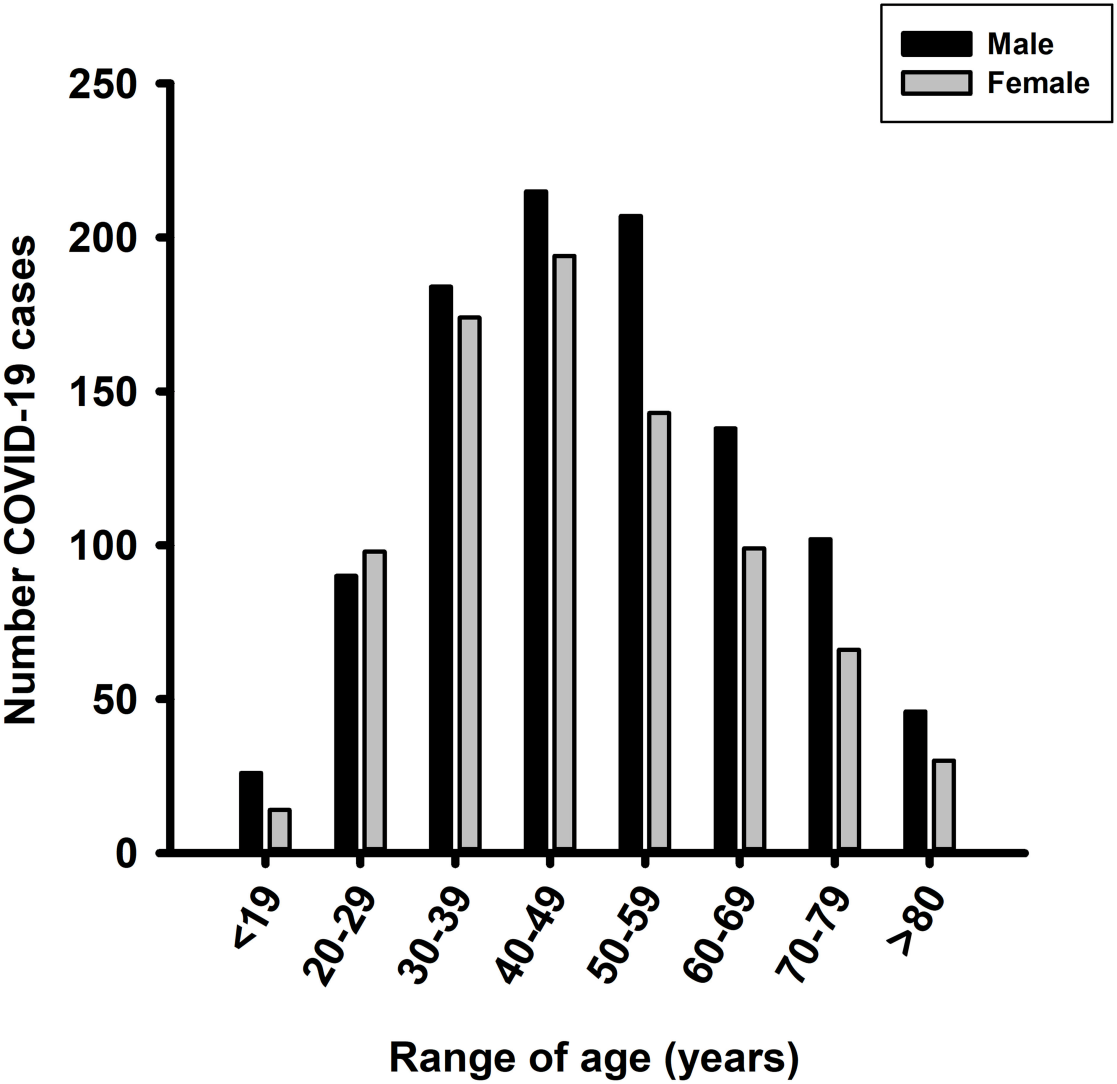
Distribution of COVID-19 cases by age range. The 1,826 COVID-19-positive patients were divided by age range.

From February 28 (date of the first case of COVID-19 registered in Sinaloa), 4,040 patients with symptoms related to COVID-19 were included in this study in which 49.8% were female and 50.2% were male. The mean age of male and female patients was 46.5 and 43.9 years, respectively (*p* < 0.001). The prevalence of SARS-CoV-2 was 45.2%, 0.6% influenza H1N1, and 0.6% other viruses (respiratory syncytial virus, influenza A H3 and B, enteroviruses, and rhinovirus). COVID-19 infection was more common in male (52.2%) than female (44.8%) patients (*p* < 0.001); additionally, the mean age of male patients was higher than that of female patients (49.7 vs. 47.3 years, respectively, *p* < 0.002). The youngest patient with COVID-19 was a 15-day-old male infant, while the oldest was a 97-year-old man. The most affected age groups were 40–49 years followed by 30–39 years and 50–59 years (Figure 1).

### Symptoms and Comorbidities in People With Respiratory Diseases

A comparison of the COVID-19 and non-COVID-19 symptoms showed that cough, fever, arthralgia, poor general health, dyspnea, chills, chest pain, diarrhea, polypnea, and cyanosis were associated with COVID-19 infection (*p* < 0.01), while myalgia, headache, runny nose, and odynophagia were predominantly associated with non-COVID-19 infections (Table 1). The remaining symptoms were found in similar proportions in all patients. Moreover, hypertension (28.3%), obesity (21.7%), and type 2 diabetes (17.8%) were the chronic diseases more prevalent in COVID-19 patients (*p* < 0.001), whereas asthma was more common in non-COVID-19 patients (6.2%, *p* < 0.001; Table 2).

**Table 1:** Clinical characteristics related to COVID-19 and non-COVID-19 patients.

**Symptoms**	**COVID-19**	^*^Non-COVID-19	***p*-value**
	**% (1,826)**	**% (2,214)**	
Cough	82.8 (1,511)	75.4 (1,669)	<0.001
Fever	79.0 (1,441)	61.8 (1,368)	<0.001
Headache	75.8 (1,381)	76.3 (1,686)	0.71
Myalgia	58.5 (1,057)	76.3 (1,082)	<0.001
Arthralgia	55.3 (55.3)	45.3 (993)	<0.001
Poor general health	51.5 (51.5)	40.2 (881)	<0.001
Dyspnea	45.6 (832)	31.8 (703)	<0.001
Chills	39.3 (711)	31.6 (693)	<0.001
Sudden onset of symptoms	38.2 (662)	32.5 (618)	<0.001
Irritability	35.8 (653)	35.5 (719)	0.02
Odynophagia	35.6 (643)	37.3 (816)	0.25
Chest pain	34.4 (623)	28.6 (626)	<0.001
Runny nose	31.7 (570)	34.0 (746)	0.1
Diarrhea	18.9 (345)	12.2 (270)	<0.001
Polypnea	15.9 (288)	11.2 (246)	0.001
Abdominal pain	11.3 (204)	10.6 (232)	0.48
Conjunctivitis	8.4 (152)	9.8 (214)	0.14
Vomiting	7.6 (138)	5.9 (130)	0.03
Cyanosis	2.9 (53)	1.7 (38)	0.01

* *Non-COVID-19 includes others virus and those with negative real-time RT-PCR test results. Pearson's chi-squared test was performed with Bonferroni correction to check for statistical significance. p <0.002*.

**Table 2:** Underlying diseases related to COVID-19 and non-COVID-19.

**Underlying diseases**	**COVID-19**	^*^Non-COVID-19	***p-*value**
	**% (1,826)**	**% (2,214)**	
Hypertension	28.3 (517)	18.1 (400)	0.001
Type 2 diabetes	17.8 (324)	11.1 (245)	0.001
Obesity	21.7 (395)	18.0 (398)	0.003
Smoking	6.5 (119)	7.3 (162)	0.31
Cardiovascular disease	5.0 (91)	3.8 (84)	0.06
Chronic kidney disease	3.2 (58)	2.3 (51)	0.08
COPD	2.9 (52)	3.0 (66)	0.43
Immunosuppression	2.1 (38)	2.3 (50)	0.7
Asthma	2.7 (49)	6.2 (137)	0.001
HIV	0.5 (10)	0.7 (15)	0.6

*COPD, chronic obstructive pulmonary disease; HIV, human immunodeficiency virus*.

* *Non-COVID-19 includes other viruses and those with negative real-time RT-PCR test results. Pearson's chi-squared test with Bonferroni correction to check for statistical significance. p < 0.002*.

### Clinical Outcomes

**Figure 2:**
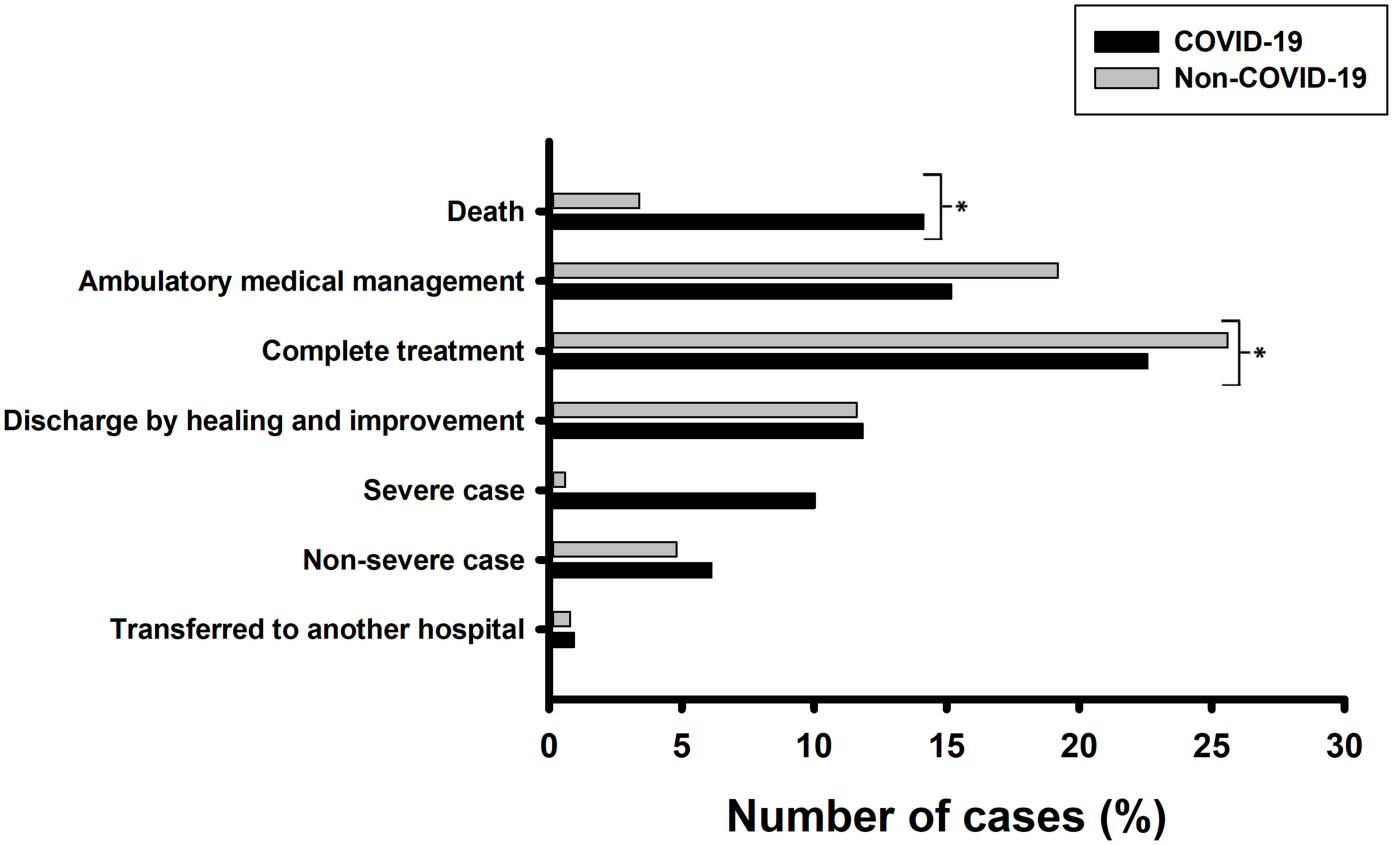
Monitoring of patient evolution. A total of 1,826 COVID-19 patients and 2,214 non-COVID-19 patients were analyzed. Pearson's chi-squared test was performed with Bonferroni correction to check for statistical significance. *p < 0.008.

Disease progression was classified as follows: (i) if the patient continued with treatment, (ii) if the patient was transferred to another hospital, or (iii) if the patient died. Comparisons showed that a higher proportion of non-COVID-19 patients were discharged than COVID-19 patients (23.1 vs. 11.4%, *p* <0.001), while a greater number of patients with COVID-19 infections died than those with non-COVID-19 infections (14.1 vs. 3.4%, *p* < 0.001; Figure 2).

### Occupational Risk for SARS-CoV-2 Infection

**Figure 3:**
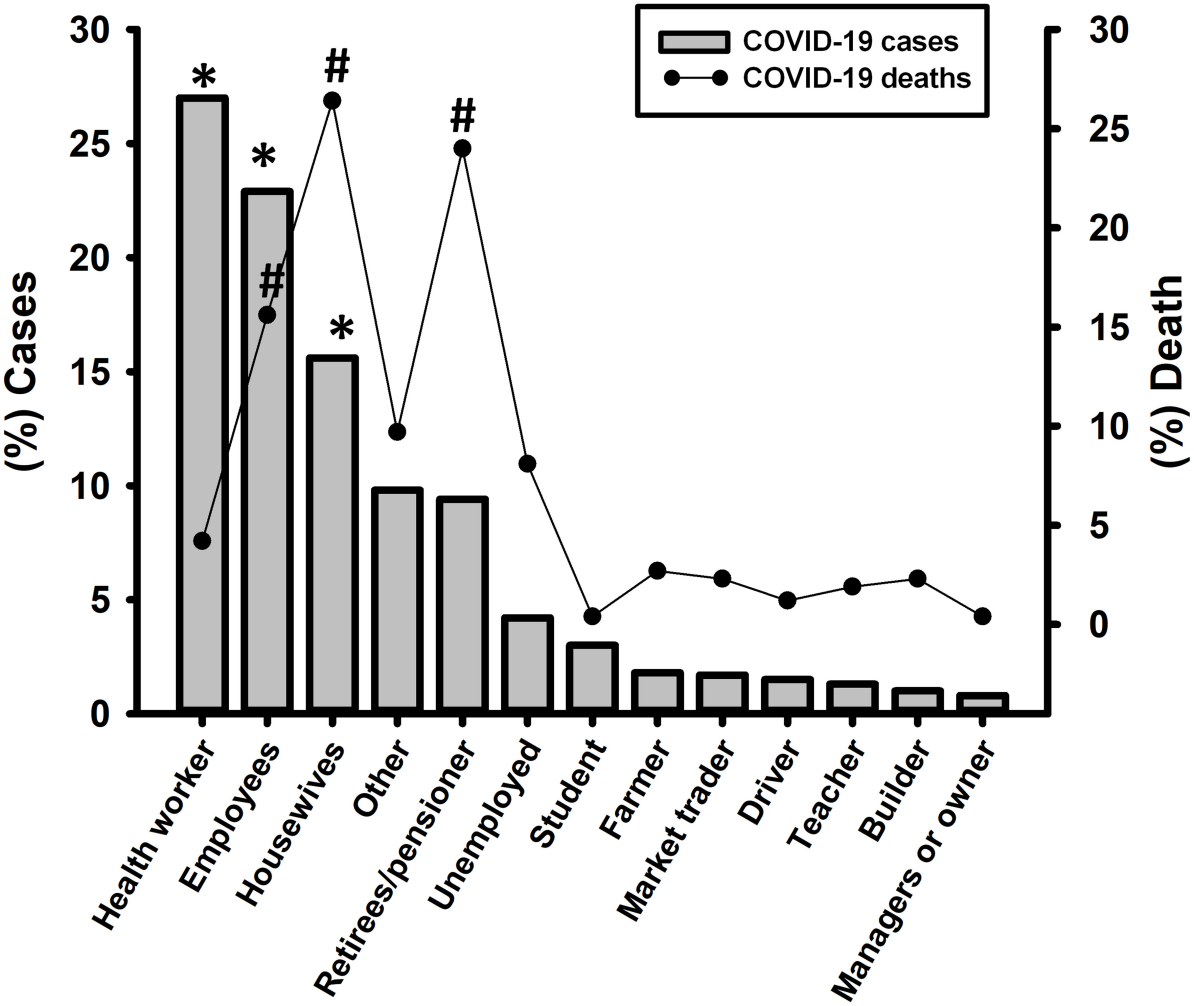
Distribution of COVID-19 cases and deaths depending on the occupation of COVID-19 patients. Pearson's chi-squared test was performed with Bonferroni correction to check for statistical significance. *p < 0.003 in COVID-19 cases; #*p* < 0.003 in COVID-19 deaths.

We found that healthcare workers, including nurses, lab workers, doctors, dentists, and workers from hospital-support areas, were the most infected (27%). Further, other employees (any employment not mentioned above, 18.5%) and housewives (15.6%) were more infected than retirees, students, farmers, drivers, teachers, merchants, and managers or business owners (*p* < 0.001; Figure 3).

### Lethality of COVID-19 in Sinaloa

Of 1,826 hospitalized patients diagnosed with COVID-19 included in this study, 57.9% were ambulatory, and 42.1% needed hospitalizing. The general lethality rate was 14.1% (258/1,826): 6.6% (17/258) were ambulatory, and 93.4% (241/258) were hospitalized patients. Overall, 67.4% of deaths occurred in male and 32.6%, in female patients (*p* < 0.001). With respect to age, lethality in COVID-19 patients ranged from 1 to 95 years. The mean age of deceased patients was 62.37 years, and the age-group most affected was 70–79 years (24%), followed by 50–59 years (23.6%).

**Figure 4:**
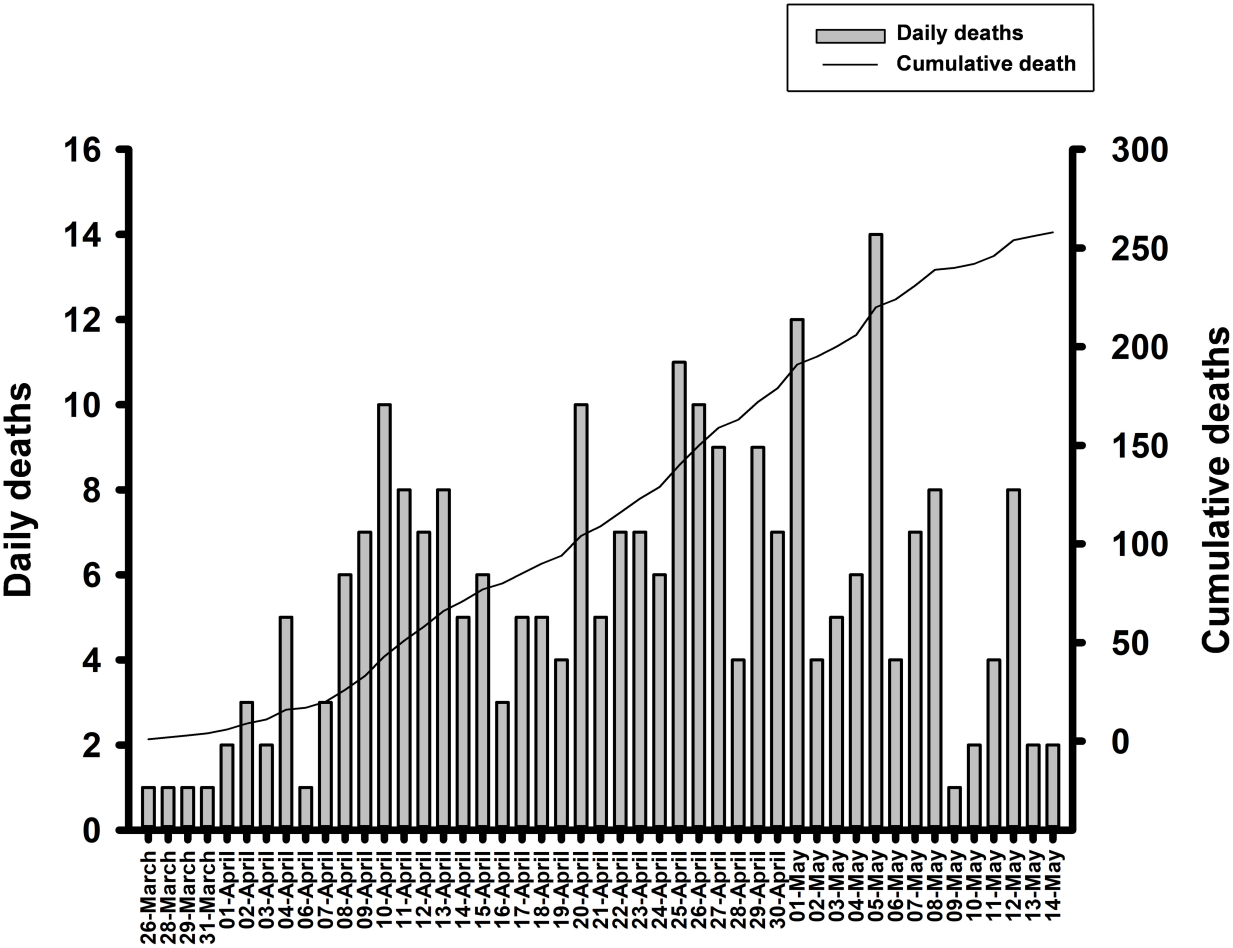
Daily deaths and cumulative deaths by COVID-19.

The first COVID-19 case in Sinaloa was registered on February 28, 2020. However, it was not until March 25, 2020, that the number of cases increased gradually to reach 10 deaths per day on April 10 (42 deaths in 16 days). Then, a slight dip occurred until April 16 (3 deaths per day) followed by a further increase to 10 deaths per day on April 26 (135 deaths in all). On May 5, a new spike was recorded with 14 deaths occurring in a day. Finally, on May 15, 258 deaths were confirmed according to the Ministry of Health report (Figure 4).

### Clinical Outcomes in COVID-19 Patients

**Figure 5:**
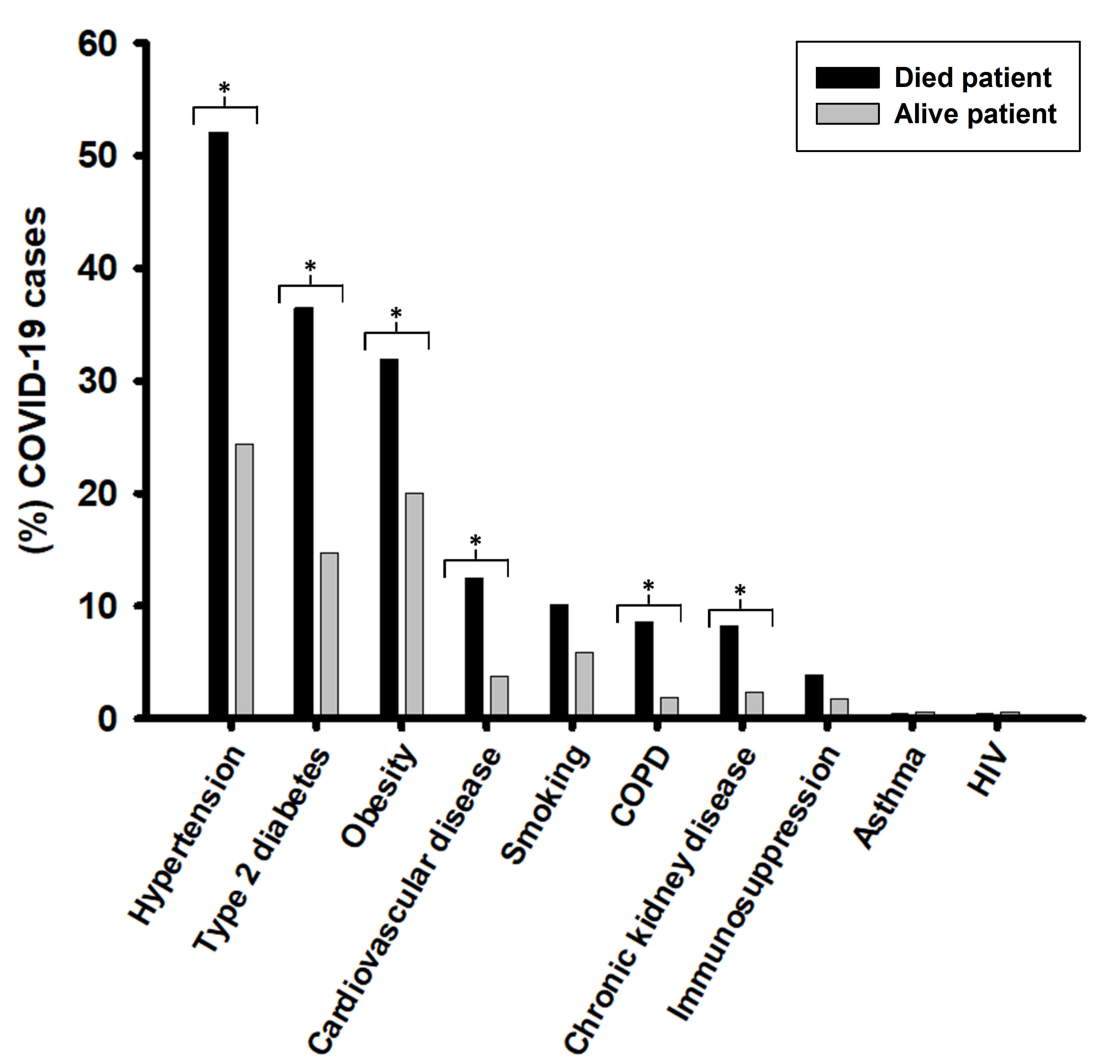
Relationship between underlying diseases and COVID-19 deaths. Pearson's chi-squared test was performed with Bonferroni correction to check for statistical significance. *p < 0.005.

We found a higher burden of the following underlying chronic diseases in deceased patients than those that survived, respectively: hypertension (52.1 vs. 24.4%, *p* < 0.001); type 2 diabetes (36.6 vs. 14.7%, *p* < 0.001); obesity (31.9 vs. 20%, *p* < 0.002); cardiovascular diseases (12.5% vs. 3.8%, *p* < 0.001); chronic obstructive pulmonary disease or COPD (8.6 vs. 1.9%, *p* < 0.001); and chronic kidney disease (8.2 vs. 2.4%, *p* < 0.001) (Figure 5). Furthermore, lethality was higher in housewives at 26.4% (68/258), followed by retirees/pensioners at 24% (62/258) and others employees with 16.3% (42/258) (*p* < 0.001), in comparison with others occupations (Figure 5).

### Influenza Vaccination and Its Relation With COVID-19 Survival

Finally, we analyzed the relationship between influenza vaccination history and COVID-19 cases who were deceased and those who survived. Of the 331 COVID-19 patients that were vaccinated against influenza, 7% (23 patients) died of COVID-19, while 93% (308 patients) survived. In the 1,406 patients that did not receive the influenza vaccine, 15% (206 patients) died, and 85% (1,200 patients) survived (*p* < 0.001).

## Discussion

Since the first reports of the novel coronavirus SARS-CoV-2 infection were confirmed in December 2019, COVID-19 spread rapidly worldwide. At present, 188 countries are affected with >5 million positive cases and >330,000 confirmed deaths worldwide. The US, UK, and Italy are the most affected countries (8). The confirmed cases and deaths by COVID-19 in Mexico continued to increase throughout the country, with Sinaloa State being the most affected state with the highest prevalence rate and lethality (9).

To the best of our knowledge, this study is the first report of the epidemiologic profile of COVID-19 in Mexico with a special focus on symptomatic patients in Sinaloa state. We found that, from February 28 to March 15, the prevalence of COVID-19 was 45% and lethality 14.1%. Men with chronic diseases and healthcare workers were the most infected groups. Finally, we found that influenza vaccination was related to COVID-19 survival when compared to unvaccinated people.

Consistent with other reports, our study also showed that men were more affected by SARS-CoV-2 infections and COVID-19-related deaths than women (10–12). This could likely be explained by differences in immune responses; recently Takahashi et al. (13) showed that males with SARS-CoV-2 infection produce high levels of pro-inflammatory innate immunity chemokines and cytokines, such as IL-8, IL-18, and CCL5, correlating with higher non-classical monocytes, while women produce a robust immune system response with T cells—in particular activated CD8 T cells. Therefore, the poor T cell responses were associated with future worsening of COVID-19 in male patients (13).

Regarding age, SARS-CoV-2 infections were more frequent in the age group of 40–49 years, similar to that reported in countries such as Spain, Canada, and The Netherlands, wherein the highest numbers of infection were in people older than 45 years (14). We also found a high proportion of positive cases in patients aged between 30 and 39 years, consistent with reports from China and Germany. In Germany, most infected people were older adolescents and younger adults. Golstein and Lipsitch (15) hypothesized that this age range was more prone to ignoring social distancing than older adults, thereby facilitating the faster spread of the virus (15).

The high proportion of COVID-19 cases among young adults could be attributed to this age group, as the majority are workers, and consequently could have less adherence to social distancing, which is similar to Germany's observations. Further, the high prevalence of chronic diseases in young adults in Mexico, such as hypertension, obesity, and type 2 diabetes, could increase the susceptibility to develop severe COVID-19 disease. This fact demonstrates that a change of lifestyle is necessary for adults and children, which includes avoidance of a western diet (diet based on obesity-inducing processed foods: foods high in saturated fats and sugars), following a healthy and well-balanced diet, and increase physical activity to prevent this phenomenon (16–18).

Moreover, the main symptoms and comorbidities related to COVID-19 reported in Mexico were similar to those reported in other countries (2, 19–21). Additionally, our data showed that healthcare workers were the most affected group because of the increased frequency of contact with suspected or infected cases of SARS-CoV-2. Previous work showed that both physical examination and nebulizer treatment increased the risk of infection among healthcare workers (22). Thus, it is necessary to improve training on the use of personal protective equipment (PPE) to reduce infections (23, 24).

The high numbers of positive COVID-19 among healthcare workers could be attributed to different facts. One of them is the lack of appropriate protective gear or the training to use it correctly. The other fact that could be contributing to the high prevalence of infection in healthcare workers is that, similarly to the general population, they also have a high proportion of chronic diseases. Vázquez-Martínez et al. (25) reported that 70% of the female staff of IMSS were obese or overweight (25). In another study, it was reported that healthcare workers in Mexico presented with diverse chronic diseases such as obesity or being overweight, high blood pressure, type 2 diabetes, and COPD (26, 27).

At present, the global lethality of COVID-19 is 6.45%; in this study, we observed a lethality rate of 14.1%, which was lower than Belgium (16.41%) and France (15.54%), similar to Italy (14.27%) and the UK (14.21%), but higher than the Netherlands (12.87%), Spain (12.19%), and Sweden (12.02%) (8) (Accessed May 23). Changes to this rate in each country depend on their epidemiologic profile (mean age, population structure, and the burden of chronic diseases), availability of health services, and adherence to the guidelines to reduce virus transmission (28). Moreover, the presence of genomic variants of SARS-CoV-2 in each country could impact COVID-19 survival (29).

Consistent with other studies, patients >65 years and with underlying chronic diseases such as hypertension, obesity, and type 2 diabetes showed a strong correlation with COVID-19 lethality (30–32). The occupations with a higher lethality rate were housewives, and retirees/pensioners. Age could be more related to COVID-19 mortality than occupation.

Moreover, we found a relation of lower risk of COVID-19 severe infection and mortality in COVID-19 patients vaccinated against influenza than those who were unvaccinated, which is consistent with previous studies (33, 34). The relation of lower risk of COVID-19 severe infection and influenza vaccine could be related to some features shared by both viruses, e.g., both viruses enter the pulmonary cells through ACE-2 receptors, and the hemagglutinin-esterase protein is very similar in both viruses. Furthermore, the spike protein has similar features to the class 1 viral membrane fusion protein of the influenza virus (35–38). Due to these similarities, the immune system response induced by the influenza vaccine could be beneficial to COVID-19 patients. Influenza vaccines also can protect against other viruses such as parainfluenza, RSV, and non-influenza virus coinfections. On the other hand, an influenza vaccine could increase the risk of other viruses, such as metapneumovirus and other coronaviruseses, by virus interference (39); however, more studies are needed to corroborate this observation.

To our best knowledge, this is the first paper to have identified the main epidemiological and clinical characteristics of patients infected with COVID-19 in one of the most affected states of México. We also highlighted the situation of healthcare workers in relation to the spread of COVID-19 in Mexico. Last, we showed a correlation between influenza vaccines and the survival of patients infected with COVID-19 in the Mexican population.

The limitation of this study is the sample size and the asymptomatic patients were not included, this study was based on patients presenting to the aforementioned hospitals only. Hence, patients attending private clinics/laboratories, those with suspected infections that did not get tested were not considered.

## Conclusions

This study provides evidence regarding the high prevalence of COVID-19 disease in symptomatic patients from healthcare institutions in Sinaloa, Mexico. It also identified risk groups (men aged 40–49 years, people with chronic diseases, and healthcare workers) that showed a greater propensity for being infected with SARS-CoV-2. The lethality registered in this study was high (14.1%), as patients >65 years with chronic diseases had a higher mortality rate than other demographics. Finally, we found a possible relationship between influenza vaccination and lower risk of COVID-19 severe infection.

## Data Availability Statement

The raw data supporting the conclusions of this article will be made available by the authors, without undue reservation.

## Ethics Statement

The studies involving human participants were reviewed and approved by The Ethics Committee of The Women's Hospital, Secretariat of Health (No. 202005-03). Written informed consent from the participants' legal guardian/next of kin was not required to participate in this study in accordance with the national legislation and the institutional requirements.

## Author Contributions

UA-Z, FM-V, NL-S, and AdC-R conceived and designed the study. HF-V, JV-R, JA-R, and JM-S collected the data. UA-Z, FM-V, NL-S, AdC-R, and JM-G analyzed and interpreted the data. UA-Z, FM-V, and AdC-R drafted the manuscript. JA-F, FU, SM-A, JS-C, JA-R, and AbC-R critically revised the manuscript. AdC-R, JA-F, AbC-R, and UA-Z approved the final version of the manuscript. All authors contributed to the article and approved the submitted version.

## Conflict of Interest

The authors declare that the research was conducted in the absence of any commercial or financial relationships that could be construed as a potential conflict of interest.
